# Modulation of allergic contact dermatitis via gut microbiota modified by diet, vitamins, probiotics, prebiotics, and antibiotics

**DOI:** 10.1007/s43440-023-00454-8

**Published:** 2023-02-02

**Authors:** Aneta Kiecka, Barbara Macura, Marian Szczepanik

**Affiliations:** Faculty of Health Sciences, Institute of Physiotherapy, Chair of Biomedical Sciences, Jagiellonian University Medical College, Kopernika 7a, 31-034 Kraków, Poland

**Keywords:** Antibiotics, Allergic contact dermatitis, Diet, Gut microbiota, Prebiotics, Probiotics

## Abstract

Allergic contact dermatitis is one of the most common recorded occupational diseases. There are many different substances that the skin comes into contact with on a daily basis and that can cause ACD, e.g., preservatives, surfactants, and antimicrobial agents. The development of a mouse model of ACD has provided insight into the immune mechanisms involved. Drugs used in the treatment of skin diseases have many side effects. Therefore, alternative methods of suppressing the immune response to reduce the symptoms of skin diseases are being sought. In recent years, high hopes have been placed on dietary modulation and supplementation to affect the intestinal microbial composition and promote anti-inflammatory responses. In addition, other studies have shown the crucial role of intestinal microbiota in many immune-mediated diseases. Recognition and characterization of pro- and anti-inflammatory nutrients and supplements may be crucial to support the treatment of diseases such as atopic dermatitis, acne vulgaris, psoriasis, and allergic contact dermatitis.

## Introduction

Atopic dermatitis (AD) is a common clinical example of an immune-mediated disease that is affected by environmental factors. AD has become a considerable clinical problem, having nearly tripled in prevalence over the last 30 years in developed countries. The disease afflicts 15–30% of children and about 2–10% of adults [1]. Yet another form of skin disease with an underlying immune-mediated hypersensitivity reaction is contact hypersensitivity (CHS) which is a mouse model of allergic contact dermatitis (ACD) in humans, which is a form of contact dermatitis (CD) and is the most common occupational disease. [2].

**Fig. 1:**
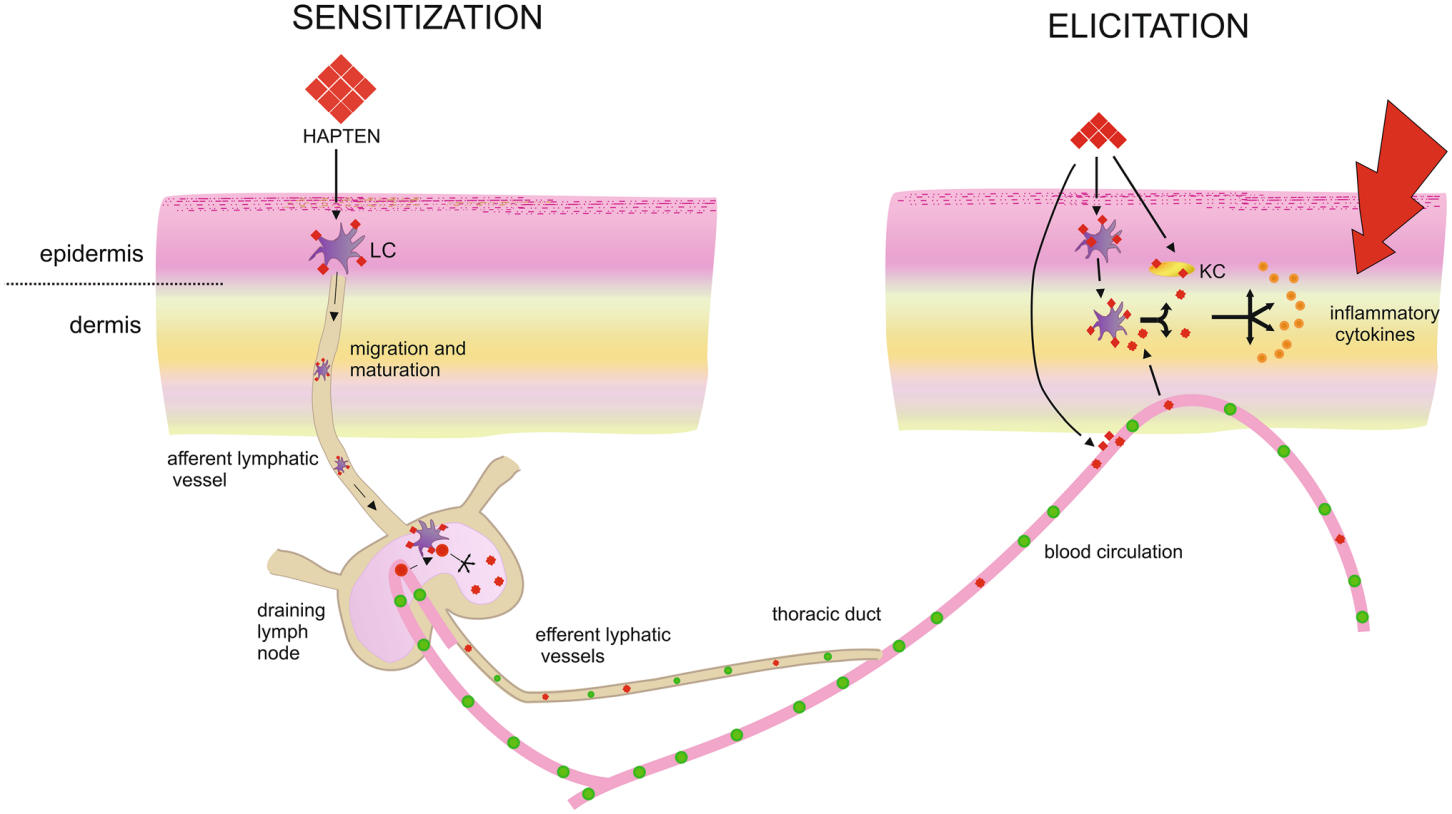
Allergic contact dermatitis (ACD). During the induction phase (sensitization), skin contact with a hapten triggers the migration of epidermal Langerhans cells (LC) via the afferent lymphatic vessels to the skin-draining lymph nodes. Haptenized-LC home into the T cell-rich paracortical areas. T cells that specifically recognize allergen–MHC molecule complexes (hapten-specific T cells) expand abundantly and generate effector and memory T cells, which are released via the efferent lymphatics into circulation. The effector T cells with their newly acquired homing receptors can be recruited locally at the site of secondary hapten challenge (elicitation phase). Due to their lower activation threshold, hapten-specific effector T cells are triggered by various haptenized cells, including LC and keratinocytes (KC), to produce pro-inflammatory cytokines and chemokines. Thereby, more inflammatory cells are recruited further amplifying local inflammatory mediator release. This leads to a gradually developing eczematous reaction, reaching a maximum within 18–48 h, after which response successively declines

Contact dermatitis can be divided into irritant contact dermatitis (ICD) and ACD. The CD accounts for the vast majority of occupational skin diseases, especially in occupations involving frequent skin contact with irritants and contact allergens. Contact dermatitis, both of allergic and irritant etiology, is the most common occupational disease in the United States [3]. ACD accounts for 20% of all registered occupational diseases in the United States [3–6]. Although ICD and ACD have similar clinical symptoms, the pathophysiology of these diseases is different. ICD involves a non-allergic response to skin irritating stimulus that disrupts the skin barrier. In ICD, the rash appears in areas of contact with the irritating stimulus. ICD is caused by substances such as acids, alkalis, soaps, detergents, among others. Reaction to the stimulus is related to the onset of inflammation and the release of pro-inflammatory cytokines from keratinocytes [7]. ACD is a type IV hypersensitivity reaction induced by contact allergens, which are small chemical molecules called haptens (Fig. 1) [8]. Out of more than 6 million chemicals present in the environment, nearly 3000 are known to cause ACD [9, 10]. There are many different substances that the skin comes in contact with on a daily basis that can cause ACD e.g., preservatives, surfactants, and antimicrobial ingredients [11]. There is a continuous search for causes and therapies for ACD. The development of CHS, which is a mouse model of human ACD has provided insight into many of this disease’s mechanisms and gives hope for finding further possible causes and possible effective therapies [12].

It has been shown that diet and diet-associated changes in the gut microbiota composition can affect the development and course of CHS [13]. Interestingly, there are reports showing that the gut microbiota can promote the development of various T cell populations with different functions such as Th17 and Treg cells [14]. Furthermore, consuming antioxidant-rich foods may moderate the immune response [15]. Additionally, animal experiments have shown that the gut microbiota is an important inducer of immune system maturation as well a regulator of immune response [16].

**Fig. 2:**
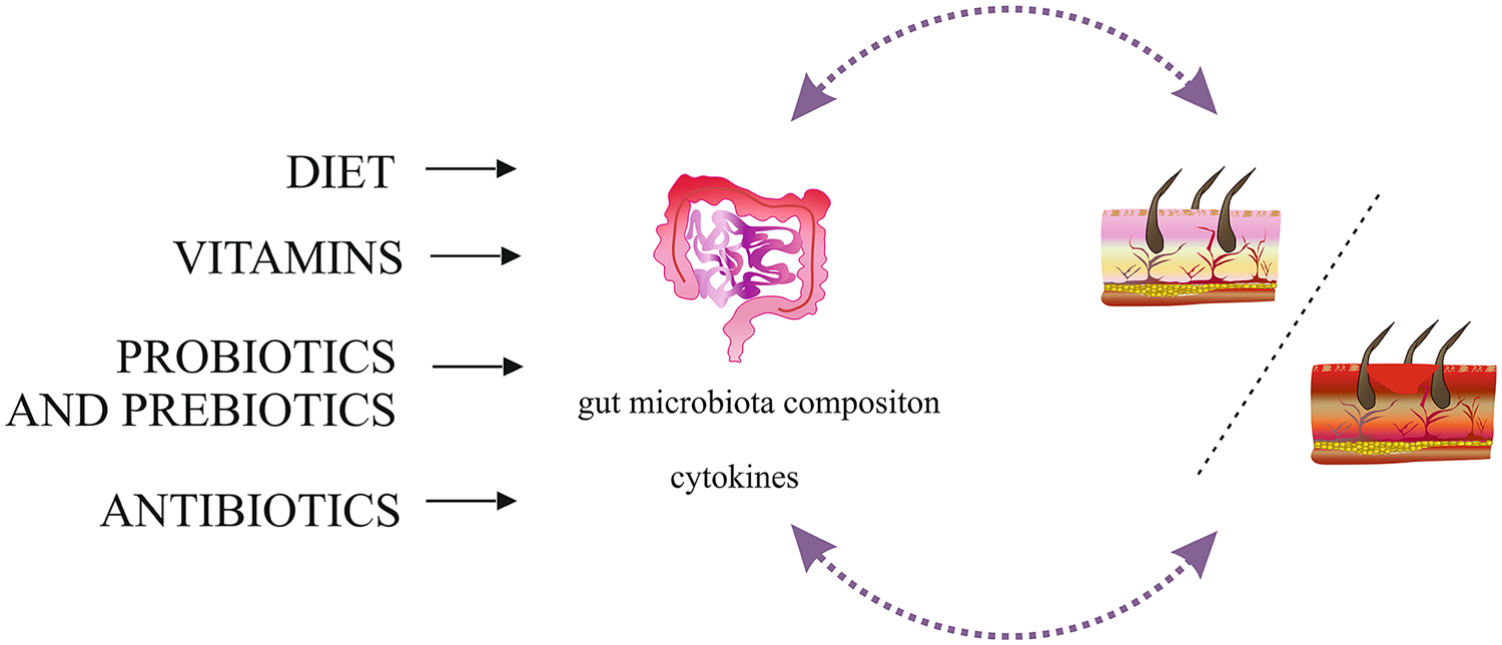
The influence of diet, vitamins, probiotics, prebiotics, and antibiotics on the regulation of CHS by modulating the intestinal microbiota. Diet, vitamins, probiotics, prebiotics, and antibiotics can promote or inhibit skin diseases including ACD. These factors can modulate cytokine production and change gut microbiota composition

The aim of this review is to describe the influence of diet, vitamins, probiotics, prebiotics and antibiotics on the regulation of CHS by modulating the intestinal microbiota (Fig. 2).

## Gut microbiota in skin diseases

Bacteria residing in the gastrointestinal tract play an important role in food digestion, xenobiotic metabolism, vitamin production, resistance to infection and maintenance of immune homeostasis [17]. The intestinal microbiota is involved in the absorption of iron, magnesium and calcium, and regulates fat storage [18]. The intestinal microbiota has been shown to be associated with the production of short-chain fatty acids (SCFAs) such as acetate and butyrate, which are known to be modulators of the immune response [17].

An imbalance of human gut microbiota (dysbiosis) in early childhood may be a risk factor for many diseases [19]. Changes in gut microbiota composition have recently been related to modulation of the immune response and development of numerous disorders including skin diseases such as atopic dermatitis, acne vulgaris, psoriasis or rosacea [20]. The gut microbiota may have a key role in the development of AD by regulating the maturation of immune system especially in early life [21, 22]. Various environmental factors such as antibiotics, stress, diet, and pollution affect the composition of gut microbiota, especially in early life [23]. *Clostridia* colonization of the infant intestines at 5 weeks of age has been shown to be associated with an increased risk of developing AD in the following 6 months of life [24]. Interestingly, the intestines of children who developed allergy were less frequently colonized by *Lactobacillus* lactic acid bacilli (*Lactobacillus rhamnosus, Lactobacillus casei, Lactobacillus paracasei*) and *Bifidobacterium adolescentis* during the first 2 months of life [25]. Ismail et al. observed a correlation between infant gut colonization by *Bifidobacterium catenulatum* and an increased risk of eczema [26]. Reduced relative abundance of potentially immunomodulatory intestinal bacteria has been suggested to be associated with excessive production of pro-inflammatory cytokines and subsequent development of eczema.

Additionally, lower numbers of *Ruminococcaceae* species have been observed in the intestines of children with eczema [27]. Studies show that adults diagnosed with atopic dermatitis have more gut *Clostridium spp.* and fewer fecal *Bifidobacterium spp.* than healthy individuals [28–31]. Penders et al. showed that gut colonization of *L. paracasei* reduced the risk of developing AD [32]. It is still unclear whether changes in the gut microbiota composition affect the development of AD, thereby regulating the immune system and disrupting the intestinal epithelial barrier, and how the presence of specific bacteria can regulate AD [33]. De Pessemier et al. showed that there may be a relationship between psoriasis and changes in β-diversity of intestinal bacteria, while acne vulgaris shows a decrease in *Firmicutes* and an increase in *Bacterioides* [20]. There are few reports defining the gut microbiota in contact dermatitis. Most of the papers focus on an animal model of CHS and identify dysbiosis as a potential source regulating skin inflammation. The mechanisms and bacteria that influence the course of skin disease are still being sought.

## Diet

The topic of diet and dermatitis has been studied for decades. Many patients with chronic dermatitis and many parents of children with AD wonder if the diet may cause or exacerbate dermatitis. It has been suggested that in some AD patients, certain foods may lead to an exacerbation of dermatitis [34]. Sampson et al. showed that AD is alleviated when foods that cause immediate allergic reactions are eliminated from the diet [35]. Dermatologists denied for years that there was a link between acne and diet, relying on studies from the 1960s. Back then, several research groups studied the relationship between chocolate consumption and acne. The largest study involving 65 patients compared the effects of eating chocolate and a placebo for a period of four weeks. There were no differences in acne severity between the study group and placebo. Many researchers have concluded that diet does not affect skin disease. However, researchers have recently re-examined this issue and found methodological flaws in previous studies. In fact, more recent data have provided strong support for the notion that a specific diet may be a potential cause of exacerbating or alleviating acne symptoms [36]. The role of certain foods, such as dairy products, as well as a high glycemic load diet in acne has been confirmed. It has also been proven that dietary changes can help inhibit skin aging. A specific diet can also be an important aspect of preventing associated systemic diseases [37]. Nutritional imbalance in the form of deficiency or excess of specific nutrients and toxic components can disrupt skin balance [38, 39]. For example, a low-calorie and low-protein diet is recommended for the treatment of psoriasis. The attenuation of psoriasis symptoms is due to a reduction in total protein intake, which leads to reduced epithelial proliferation and lowers polyamine levels [40].

Most studies investigating the effects of diet on contact hypersensitivity are based on mouse studies. It has been proven, among others, that the consumption of specific foods can regulate hapten sensitivity. This chapter describes unsubstantiated associations of specific nutrients on the regulation of immune responses in the CHS. Unfortunately, much of the data are inconsistent, and while some researchers show benefits of specific nutrients in the diet, others show that they may exacerbate inflammation.

Medications used to treat skin conditions have many side effects including skin atrophy, sunspot, stretch marks, muscle weakness, headaches, and high blood pressure. Therefore, natural ways to weaken inflammation are constantly being sought, including the elimination of foods from the diet that may promote inflammation.

### Soybean and its derivatives

There are many reports on the health-promoting effects of soybean and its derivatives [41]. Soy-based foods are rich in active phytochemicals such as soy saponins (SS) and soy isoflavones (SI) [42, 43]. The SSs are proven to benefit health through their antioxidant and anticancer properties, inhibition of inflammation, and cardiovascular protection [44, 45]. Soy saponins inhibit the release of pro-inflammatory mediators such as TNF-α and monocyte chemoattractant protein-1 (MCP-1) and inhibit the activation of nuclear transcription factor NF-kappa β (NF-κB) in lipopolysaccharide-stimulated peritoneal macrophages [46, 47]. Further studies show that feeding mice a diet rich in SS or SI inhibits the CHS response. It has been observed that there is a reduction in tissue swelling and inhibition of Gr-1^+^ cell infiltration and a decrease in the activity of pro-inflammatory mediators [48, 49]. It has been observed that even low-dose SS supplementation can inhibit the CHS response [47–52]. Lee et al. showed that dietary SS enrichment inhibited the production of pro-inflammatory cytokines (TNF-α and IL-1β), inflammatory mediators (NO and PGE2) and inflammatory enzymes (COX-2 and iNOS) in LPS-stimulated peritoneal macrophages. In addition, a decrease in myeloperoxidase and NF-κB activity was observed, which was associated with possible mitigating effects of SS on colitis induced by 2,4,6-trinitrobenzene sulfonic acid (TNBS) in mice [53]. The DNA microarray analysis showed that the expression of *Ccl24, Xcl1 Ifng* and *Ccl17* genes were downregulated to a greater extent in the soy-fed mice group than in the control group [48].

It has been shown that the consumption of soy products can alter the composition of gut microbiota [54, 55]. For example, substances such as glycine, β-conglycine, raffinose, stachyose, sucrose, fructose, glucose, galactose, and other mono- and oligosaccharides are present in soy milk, which can be used by gut bacteria and may change the gut microbiota composition [56]. Fernandez-Raudales et al. studied the effects of soymilk consumption on the gut microbiota of overweight men and found an increase in beneficial bacteria in the intestine. They reported that these changes were related to a reduced risk of obesity and other metabolic diseases [54]. Soy compounds have been shown to exhibit poor intestinal absorption. Therefore, their effect on the CHS response is likely indirect and may be due to their modulating properties of the gut microbiota [57]. Nagano et al. in mice orally administered SS before inducing a CHS response, observed a decrease in the number of bacteria belonging to the *Lachnospiraceae* and *Desulfovibrio* families (*p* < 0.05) and a slight increase in the number of bacteria belonging to the *Porphyromonadaceae* family in the intestine compared to a group of mice sensitized to hapten but fed a conventional diet. These changes in the composition of gut microbiota in the group of mice fed the SS-enriched diet correlated with the inhibition of the CHS response (*p* < 0.01). Mice also showed an inhibitory effect of soy milk feeding on the CHS, which was investigated by measuring ear swelling. Hapten sensitized mice treated with soy milk showed inhibition of the CHS and reduced ear swelling (37%, *p* < 0.01). In the gut of these mice, the relative abundance of *Lactobacillaceae* was significantly lower in control sensitized mice compared with mice receiving soy milk orally prior to CHS induction (*p* < 0.05). The relative abundance of *Lachnospiraceae* (*p* < 0.05), *Ruminococcaceae* (*p* < 0.01), *Desulfovibrionaceae* (*p* < 0.05) and *Rhodothermaceae* (*p* < 0.01) was significantly increased in the CHS mice compared to non-CHS mice treated with soy milk. It was also shown that the relative abundance of *Ruminococcaceae* in control hapten-sensitized mice was significantly higher than in hapten-sensitized mice treated with soy milk (*p* < 0.01). Despite extensive research on the health benefits of soy, the complexity of soy components makes it difficult to identify the exact active ingredient or compound responsible for a particular health-promoting effect and specific biological activity [50].

### Polyphenols

Polyphenols and polyphenol-derived compounds from plants are common causes of skin reactions leading to ACD and/or skin irritation. Most contact allergens of polyphenolic origin are reactive quinones or polyphenols that are oxidized to quinones in the skin. However, there are reports that some polyphenols have anti-inflammatory effects and/or can inhibit T cell activation and proliferation, so they can alleviate symptoms of dermatitis and CHS [58–60]. Magrone et al. demonstrated that in vitro administration of polyphenols extracted from red grape seeds to peripheral blood cells of patients suffering from ACD to nickel can reduce the release of pro-inflammatory cytokines and nitric oxide (NO), while increasing levels of interleukin-10 (IL-10), an anti-inflammatory cytokine [61].

Nagano and Hideyuki, in a mouse model of CHS induced in BALB/c mice by dinitrofluorobenzene (DNFB) sensitization, used a diet rich in pomegranate fruit polyphenols to determine its effect on skin inflammation. They showed that a diet rich in pomegranate polyphenols reduces ear swelling and inhibits infiltration of Gr1^+^ myeloid cells into ear tissues, and that there is a reduction in CXCL2 and MCP-5 production [62]. Anderson et al. showed that polyphenol-rich black raspberry extract can inhibit CHS in mice. Interestingly, a significant reduction in the accumulation of CD11c^+^ DCs in the spleen was observed in DNFB-treated mice fed a diet supplemented with raspberry polyphenols, compared to DNFB-sensitized mice fed a control diet. Black raspberry extract reduced CD80 expression and interleukin-12 (IL-12) secretion [63]. In their studies, Ikarashi et al. showed the inhibitory effect of a polyphenol derived from *Acacia mearnsii* on CHS induced by trimellitic anhydride (TMA) administration. *Acacia* extract caused inhibition of the expression levels of TNF-α, IL-6, iNOS and COX-2. It is well known that polyphenols have a low absorption rate. Therefore, it is believed that polyphenols are unlikely to affect the skin after absorption into the body and may indirectly affect the CHS response. Polyphenols have been shown to alter the colonic microbiota [64, 65]. It was found that the abundance of *Bifidobacterium* spp. in the intestinal microbiota significantly decreased in a group of TMA sensitized mice, while an increase in *Bifidobacterium spp*. and *Lactobacillus* spp. in the intestine was observed after administration of acacia extract [66]. *Sargassum horneri* is an edible brown alga known for its beneficial biological properties, including anti-inflammatory properties. It was investigated whether polyphenol-rich extracts of *S. horneri* could suppress AD-like skin lesions in NC/Nga mice. Polyphenol-rich *S. horneri* was shown to attenuate AD-like skin lesions in NC/Nga mice by inhibiting IL-13 production by Th2 cells [67].

### High-fat diet

Obesity caused by the consumption of the western diet defined as a high dietary intake of saturated fats and sucrose and a low intake of fiber has increased enormously over the past decades. The consequences of obesity can include hyperglycemia, dyslipidemia, hyperinsulinemia, and increased adipose tissue secreting various adipokines, chemokines, and cytokines [68]. Furthermore, free fatty acid (FFA) levels have been shown to be elevated in obese patients, contributing to inflammation and insulin resistance. Recently, adipose tissue has been recognized as a multifunctional organ. In addition to its central energy storage role, it has an important endocrine function, secreting several hormones called adipokines, in particular leptin and adiponectin, which regulate not only energy metabolism but also inflammation and immune response [69]. The metabolic and immune systems are closely related and functionally dependent. Obesity leads to an imbalance in cellular immunity in both human and animal studies. This condition is also characterized by an increased number (hyperplasia) and size of adipocytes. Adipocyte hypertrophy leads to hypoxia in adipose tissue and causes dysfunction or necrosis of adipocytes. As a result, damage-related factors are released and recognized by pattern recognition receptors (PRR) [70, 71]. This contributes to the activation of inflammatory and stress responses, resulting in a chronic low-grade inflammation called “metabolic inflammation” or “metainflammation” [72, 73]. In addition, gut dysbiosis is observed among obese individuals, characterized by high *Firmicutes* and low *Bacteroidetes* [74, 75]. The dysbiosis state potentially contributes to the exacerbation of inflammation. Interestingly, obese and overweight subjects more often have ACD [76].

Katagiri et al. demonstrated that a high-fat diet (HFD) modulates skin immunity in the mouse. The levels of IFN-γ and IL-4 are reduced [69]. Rühl-Muth et al. in their study investigated the impact of HFD on the sensitization and elicitation of the CHS reaction in wild-type and TLR2/4 knock-out mice that are resistant to CHS. The authors found that the CHS reaction of wild-type mice to TNCB was increased by HFD. Interestingly, HFD feeding broke the resistance of TLR2/4 knock-out mice to TNCB. They showed a tendency to increase pro-inflammatory cytokines such as IL-1β, IL-6, and TNF-α in the serum 24 h after TNCB treatment [77].

In a study conducted by Kowalczyk et al. Rag1^−/−^ mice fed HFD for 8 weeks experienced an exacerbation of the NK-mediated CHS response as determined by measuring ear swelling compared to animals kept on a normal diet (ND). In vitro analysis showed that HFD feeding significantly increased IFN-γ and IL-12p70 levels and decreased adiponectin levels in the liver mononuclear cell (LMNC) culture supernatants. Flow cytometry analysis of LMNCs showed that HFD treatment prior to DNFB sensitization increases the percentage of NK1.1^+^ IFN-γ^+^ cell population and affects NK1.1^+^ cell development and maturation [78]. Interestingly, a study by Higashi et al. showed that HFD-fed mice exhibited exacerbation of psoriasis symptoms, and the number of neutrophils infiltrating the skin lesions was elevated. The mRNA expression of IL-17A was significantly increased in the skin of HFD mice, whereas the mRNA expression of IL-22, IL-23, and TNF-α was not enhanced. The caspase-1 and IL-1β were activated in the skin of HFD mice and their serum levels of IL-17A, TNF-α, and IL-1β were significantly increased. It has also been shown that hyperlipidemia is also involved in the development and progression of psoriasis through systemic inflammation and activation of inflammasome [79].

## Vitamins

The role of vitamins in skin diseases such as porphyria cutanea, malignant melanoma, acne, and atopic dermatitis has been previously observed [80]. However, the data obtained on the effect/mechanism of vitamins on these diseases is poorly understood. Moreover, there is sparse knowledge about the influence of vitamins on ACD and CHS.

### Vitamin C

Vitamin C (ascorbic acid, ascorbate), a simple low molecular weight carbohydrate, has been shown to be involved in skin barrier formation and collagen production in the dermis and has physiological roles in the skin to counteract skin oxidation, anti-aging, as well as in cell growth and differentiation signaling pathways that are associated with the occurrence and development of various skin diseases [81]. Vitamin C can inhibit AD by promoting keratinocyte differentiation and maintaining the skin barrier [82], and in the case of melanoma, it has an effect on inhibiting HIF-1 alpha transcriptional activity, thereby preventing tumor growth and metastasis [83]. One study showed that topical application rather than supplementation of vitamin C in patients with ACD can mitigate skin inflammation [84].

### Vitamin E

Another vitamin that may be important for skin disease is vitamin E. It is an important fat-soluble antioxidant with over 50 years of use in dermatology. In fact, it is an important ingredient in many cosmetic products. It protects the skin from various harmful effects of sunlight by acting as a “sweeper” of free radicals. Experimental studies suggest that vitamin E has anticancer and photoprotective properties. It can be used, among others, in pustula subcornealis dermatoses [85]. Vitamin E (α-tocopherol) intake also affects the composition of gut microbiota an important modulator of the immune response [86]. Tsoureli-Nikita et al. in 96 patients with AD who were treated by oral administration of vitamin E (400 IE/day) for 8 months observed remission of AD and a 62% decrease in serum IgE levels 1005 to 490 IU/ml [87]. Jaffary et al. showed in 70 patients with mild to moderate AD, that daily supplementation with 400 IU of vitamin E resulted in attenuation of AD symptoms after 4 months compared to placebo (*p* < 0.05) [88]. Vitamin E is able to neutralize free radicals on its own and modulate many signaling pathways including PPAR, STAT6, NF-κB, and Nrf2. It can also regulate many cytokines (IL-1β, IL-4, IL-6, IL-13, TNF-α, TGF-β and G-CSF), kinases (ERK, MAPK, PI3K, and PKC) and enzymes (Cat, GPx, SOD, HO-1, COX-2, 5-LO, and PLA2) that are involved in both inflammation and oxidative stress [89]. Vitamin E supplementation to healthy volunteers resulted in the inhibition of various pro-inflammatory cytokines released by monocytes such as IL-1β, IL-6, and TNF-α [90]. In a randomized clinical trial among patients suffering from allergic dermatitis, the administration of 400 mg of α-tocopherol daily for 60 days resulted in elevated plasma α-tocopherol levels and a 35.7% reduction in skin inflammation [91]. Kuriyama et al. showed that 20% vitamin E ointment suppresses contact dermatitis via stabilizing keratinocytes [92]. Ikarashi et al. show that vitamin E can modulate the immune response through scavenging reactive oxygen species [93].

### Vitamin D

Vitamin D plays an important role when it comes to the skin. Keratinocytes are not only the source of vitamin D, but also respond to its active form [94]. According to Litonjua and Weiss, vitamin D not only helps regulate calcium, blood pressure, and electrolyte levels, but is also essential in regulating immune response. Vitamin D deficiency is a contributing factor to the increased incidence of autoimmune diseases such as rheumatoid arthritis (RA), systemic lupus erythematosus (SLE), multiple sclerosis (MS), type 1 diabetes (T1D), inflammatory bowel disease (IBD), and other autoimmune diseases [95, 96]. Vitamin D has immunomodulatory properties, including effects on T lymphocytes, B lymphocytes and dendritic cells. Vitamin D use has been shown to lead to the development of DCs with tolerogenic properties. Th17 lymphocytes, a subset of CD4^+^ T cells, have been shown to be crucial in various autoimmune diseases. Interestingly, vitamin D has been shown to inhibit autoimmunity and tissue destruction by suppressing the Th17-mediated immune response. In addition, vitamin D increases levels of anti-inflammatory cytokines such as IL-4, IL-5, IL-10, TGF-β as well as by inhibiting the production of pro-inflammatory cytokines such as IL -2, IL-3, IFN- γ, TNF-α [95]. Vitamin D also has a protective role in allergic diseases. It has the ability to inhibit both Th1 and Th2 type responses by inhibiting IL-12 production as well as IL-4 and IL-4-induced IL-13 expression. Moreover, Vitamin D promotes development of CD4^+^ T regulatory (Treg) cells that effectively inhibit abnormal immune response in autoimmunity [ref]. Vitamin D has a potent antiproliferative effect on CD4^+^ T lymphocytes, along with the ability to inhibit T lymphocyte function, both directly and by affecting antigen-presenting cells (APC). Vitamin D and its receptor (VDR) are essential for the development of natural killer (NK) cells and the expression of IL-4 and production of IFN-γ. NK cells contribute to the development of allergic airway inflammation mediated by T cells and are capable of producing numerous pro-inflammatory cytokines such as IFN-γ, TNF-α, GM-CSF and MIP-1. [96]. Vitamin D may also modulate the composition of gut microbiota and alleviate intestinal dysbiosis among patients with autoimmune disorders [97]. Importantly, vitamin D deficiency in the intestine is associated with inflammation, colonic shortening, impaired mucosal structure, and inflammatory cell infiltration. Vitamin D deficiency is also associated with a reduction in the mucus layer through a change in microbial composition characterized by an increase in *Akkermansia muciniphila*, a bacterium that degrades gut mucin [98]. The study conducted by Cantarel et al. showed that among MS patients, supplementation with 5000 IU of vitamin D daily for 90 days increased the abundance of *Faecalibacterium* and *Coprococcus*, which produce butyrate an anti-inflammatory SCFA, in the gut [99]. Vitamin D has shown to inhibit Th1 and Th2 type responses by suppressing the production of IFN-γ and IL-4 [96]. Vitamin D also regulates AMP synthesis in the skin and exerts an immunosuppressive effect on the skin by reducing antigen presentation either directly affecting LC or indirectly by regulating cytokine production by keratinocytes [100, 101]. Although there are no clinical studies evaluating a potential link between vitamin D and CD in humans, there is one study employing mice that evaluates this potential link. Malley et al. compared the CHS responses of mice with normal vitamin D levels and vitamin D deficient mice. Vitamin D-deficient male mice showed a significantly increased CHS response compared to males with normal vitamin D levels. Interestingly, there were no significant differences in CHS reactions in female mice between the vitamin D-deficient and normal vitamin D groups [102]. To date, there are no studies suggesting that vitamin D may actually play a role in ACD in humans. Therefore, vitamin D supplementation in ACD is not recommended and requires clinical trials [103].

## Probiotics and prebiotics

Around the end of the twentieth century, the first studies appeared showing the potential beneficial effects of probiotics in alleviating inflammation. Probiotics are microorganisms that affect the gut microbiota and have been the focus of research by scientists due to their possible therapeutic properties of AD [104–107]. *L. plantarum* LM1004 has been shown to have the ability to inhibit Th2-mediated immune response, which is crucial for allergic reactions, while activating Treg and galectin-9 Th cells that have immunoregulatory activity [108]. Boyle et al. showed by analyzing 12 randomized trials that probiotics are not an effective treatment for eczema, and probiotic treatment is associated with a low risk of side effects [109]. In contrast, Osborn et al. showed a reduction in AD with probiotic supplementation in infants at high risk for the disease [110]. Weston et al. studied 56 children aged 6–18 months with moderate to severe AD. Children were given a probiotic (1 × 10^9^ *Lactobacillus fermentum* VRI-033 PCC) or an equivalent volume of a placebo, twice daily for 8 weeks. The final evaluation was conducted after 16 weeks. Supplementation with the probiotic *L. fermentum* VRI-003 PCC has been shown to be beneficial in inhibiting AD among young children with moderate to severe skin disease [111]. Furthermore, oral administration of lactic acid bacilli to pregnant women has been shown to protect high-risk children from AD [112, 113]. In another study, *Lactobacillus sakei* (WIKIM30), a Gram-positive facultative anaerobic bacterium isolated from kimchi—a Korean fermented plant food was shown to inhibit serum IgE and IL-4 production. Treatment of mice with WIKIM30 reduced tissue swelling and decreased CD4^+^ T cells and B lymphocytes, as well as Th2 cytokine expression. Oral administration of WIKIM30 also modulated the gut microbiota, which may influence the allergic immune response in AD mice. In sensitized mice, a reduction in the abundance of *Ruminococcus* was observed, which was increased after WIKIM30 treatment. The relative abundance of *Artromitus* and *Ralstonia*, which was elevated in AD, was reduced by WIKIM30 treatment, indicating that an increase in the abundance of these two genera may be positively correlated with Th2-related responses in AD [114].

There are many animal studies supporting the protective role of probiotics in skin diseases, including CHS. Chapat et al. showed that oral administration of *Lactobacillus casei* DN-114 001, a strain used to prepare fermented milk, reduced the CHS response induced by DNFB in C57BL/6 mice. Probiotics reduced the hapten-specific response mediated by IFN-γ-producing CD8^+^ Tc1 effector cells. Probiotic supplementation reduced IFN-γ production upon re-stimulation with hapten [115]. In a study conducted by Sasajima et al. oral administration of *B. pseudolongum* resulted in decreased CHS responses only in the initial phase. It is inferred that the proliferation of *B. pseudolongum* in the gastrointestinal tract is partly responsible for the reduction of DNFB-induced CHS response in mice, which may be mediated by the modulation of produced cytokines [116]. Similar results were observed when *B. longum 51A* was administered to oxazolone (OX) sensitized mice. A reduction in ear and skin thickness as well as a lack of leukocyte infiltration was observed. However, it was also observed that administration of inactivated *B. longum 51A* had no effect on the inhibition of the CHS response, suggesting that the bacteria must be alive to be effective. Given that *B. longum 51A* is an acetate producer, mice were given acetate intraperitoneally, which also reduced ear and skin swelling [117].

Prebiotics are defined as non-digestible food components that selectively stimulate the growth or activity of one or a specific number of bacterial genera in the colon that benefit the host’s health. Prebiotics such as inulin, fructooligosaccharides, lactulose, or derivatives of galactose and β-glucans can be introduced artificially into foods to improve nutritional and health values. They are nutrients for probiotics, stimulate their growth and, unlike probiotics, there are no microorganisms in their composition [21]. Prebiotics have shown similar inflammation-alleviating properties. Prebiotic carbohydrates are considered to ferment in the cecum and colon, leading to an increase in fermentable bacterial strains and their metabolites in this part of the gastrointestinal tract. Hansen et al. fed mice with the prebiotic xyloligosaccharide (XOS) and found that the prebiotic diet increased the presence of *Bifidobacterium* in the intestine compared to mice fed the classical feed, with the highest percentage found in the ileum (*p* < 0.001). The expression of IL1-β (*p* < 0.01) and IFN-γ (*p* < 0.05) was shown to be significantly lower in the blood of mice fed XOS than mice fed the control diet. It has been shown that XOS feeding reduces systemic inflammation, and this effect is most likely due to increased production of short-chain fatty acids (SCFAs) as a result of increased fermentation of bifidobacteria in the intestine [118]. Laigaard et al. studied the effect of a prebiotic (XOS) on gut microbiota and otitis in an OX-induced dermatitis model in BALB/c mice. XOS-fed mice were shown to have a higher abundance of *Prevotella spp.* in the intestinal microbiota correlated with reduced CHS response [119]. Similarly, dietary supplementation with prebiotic fructooligosaccharides (FOS) was shown to reduce DNFB-induced CHS response in BALB/c mice [31]. Studies show that FOS supplementation results in higher levels of bifidobacteria in mouse feces, which may contribute to the inhibition of CHS responses [116]. It was also shown that the amount of IL-17 in the damaged ear skin was significantly lower in FOS-fed mice [120]. The effects of prebiotic fucosylated chondroitin sulfate from *Acaudina molpadioides* on modulating the gut microbiota and influencing the risk of chronic inflammation in mice fed an HFD were also evaluated. Results showed that administration of this prebiotic significantly modified the gut microbiota, including observed decreases in *Bacteroidetes*, increases in *Firmicutes* and *Lactobacillus spp.* strengthening intestinal barrier and SCFA-producing bacteria (*Lactobacillus, Bifidobacterium*, and *Lachnospiraceae* of the NK4A136 group). This modulation inhibited the inflammatory response expressed as a decrease in circulating pro-inflammatory cytokines and an increase in IL-10. All these data suggest that modulation of intestinal microbiota by probiotics and prebiotics may attenuate chronic inflammation [121].

## Antibiotics

Statens Serum Institut in Denmark has observed a steadily increasing consumption of antibiotics worldwide since 1995 [122]. Antibiotic use has been shown to modulate intestinal microbiota in both children and adults [123]. Among children born to mothers with atopic dermatitis, antibiotic use during pregnancy was associated with an increased likelihood of AD in children if antibiotics were used in all three trimesters of pregnancy [124]. McKeever et al. conducted a cross-sectional study on 24,690 children in the UK and found that antibiotics given to women during pregnancy increased the likelihood of AD in children in a dose-dependent manner [125]. The effect of oral administration of enrofloxacin on anti-OVA antibody production and cytokine synthesis has been reported in mice. It was observed that enrofloxacin treatment in adult mice leads to a significant reduction in the production of anti-OVA IgG2a, while the synthesis of anti-OVA IgE is not altered [126].

Kowalczyk et al. evaluated the effects of enrofloxacin use during pregnancy on the immune response in the CHS reactions and the composition of bacteria in the feces of offspring. Treatment with enrofloxacin during pregnancy was shown to exacerbate the CHS response in adult mice as measured by evaluating ear swelling and decreased the relative abundance of *Clostridium cluster* IV [127]. It has been shown that *Clostridium* cluster IV bacteria have potent anti-inflammatory properties because they increase intestinal TGF-β levels and promote the development of inducible IL-10-producing Treg cells [128]. Interestingly, a study in adult mice showed that oral treatment with enrofloxacin inhibits CHS and IgG1 antibody production against trinitrophenyl chloride (TNP-Cl). Further, this work shows that antibiotic administration promotes the induction of numerous regulatory cells that suppress CHS. Flow cytometry and transfer of purified cells show that antibiotic-induced suppression of CHS is mediated by TCR αβ^+^ CD4^+^ CD25^+^ FoxP3^+^ Treg, CD19^+^ B220^+^ CD5^+^ IL-10^+^, IL-10^+^ Tr1, and IL-10^+^ TCR γδ^+^ cells [126].

## Conclusion

Over the years, researchers have recognized how important the microbiota is in the pathogenesis of many diseases. The microbial composition of individual organs can influence the course of the disease. The intestinal microbiota has been shown to play a significant role in health and disease. It is sometimes referred to as the “forgotten organ.” The microbiota is involved in various metabolic functions such as fermentation and absorption of undigested carbohydrates. More importantly, the microbiota interacts with the immune system, providing signals that promote the maturation of specific immune cells and the development of the immune function. The bacterial composition of intestinal microbiota has been extensively studied in recent years by the large-scale Human Microbiome Project and MetaHIT studies. Metagenomic studies of intestinal microbiota have shown that despite considerable interpersonal variability in microbiota composition, there is a common core. It is this population-wide common core that may be an important element in the pathogenesis of many diseases. It has been shown that the composition of the intestinal microbiota can be influenced by various environmental factors such as stress, diet, antibiotics, and pollution.

Drugs used to treat skin diseases have many side effects including skin atrophy, sunspot, stretch marks, increased hair thickness, muscle weakness, headache and high blood pressure. Therefore, natural ways to alleviate inflammation are constantly being sought, including elimination of foods from the diet that may promote inflammation. There is emerging hope in using an appropriate diet to prevent or alleviate the disease’s symptoms. The term “anti-inflammatory diet” has even been introduced. Recent studies show that such a diet is being studied in diseases such as RA, Crohn’s disease and ulcerative colitis. However, it is not clear if nutrients regulate the immune system directly or indirectly by modulating the composition of intestinal microbiota.

The data included in the review show that specific nutrients including soybean and its derivatives or polyphenols, or vitamins can regulate inflammation in a mouse model of CHS. Pro-health effects of probiotics have also been reported. However, as in the case of e.g., vitamin A or soy saponin, the protective effect on the skin is dose dependent. Nevertheless, reports showing the beneficial effect of a specific diet on the alleviation of disease symptoms provide great hope for finding natural methods of treating diseases by modulating the immune system. Unfortunately, the exact pathways by which nutrients can regulate the immune response in skin diseases are still unknown. However, further research shows that this may be achieved through the modulation of intestinal microbiota. It is necessary to continue research to find all the ingredients with anti-inflammatory effects and to develop a diet that is beneficial for a given condition with potential inflammation-relieving properties and learn the exact regulatory mechanism of diet on the immune response. Moreover, studies show that probiotics, prebiotics and antibiotics can modulate gut microbiota. Probiotics are microorganisms that affect the gut microbiota and have been the focus of research by scientists due to their possible therapeutic properties of AD. Prebiotics have shown similar inflammation-alleviating properties. Prebiotic carbohydrates are considered to ferment in the cecum and colon, leading to an increase in fermentable bacterial strains and their metabolites in this part of the gastrointestinal tract. Moreover, studies show that among children born by mothers with atopic dermatitis, antibiotic use during pregnancy was associated with an increased likelihood of AD in children if antibiotics were used in all three trimesters of pregnancy. Further research on the effect of antibiotic therapy and supplementation on the intestinal microbiota and ACD is necessary.

## Data Availability

Not applicable.

## Abbreviations

ACD: Allergic contact dermatitis
AD: Atopic dermatitis
AMP: Adenosine-5′-monophosphate
APC: Antigen-presenting cell
CD: Contact dermatitis
CHS: Contact hypersensitivity
COX-2: Cyclooxygenase-2
DC: Dendritic cell
DNFB: 1-Fluoro-2,4-dinitrobenzene
FOS: Fructooligosaccharides
GM-CSF: Granulocyte–macrophage colony-stimulating factor
HFD: High fat diet
IBD: Inflammatory bowel disease
ICD: Irritant contact dermatitis
IFN-γ: Interferon- γ
IL-1β: Interleukin-1β
IL-4: Interleukin-4
IL-5: Interleukin-5
IL-10: Interleukin-10
IL-12: Interleukin-12
IL-13: Interleukin-13
IL-17: Interleukin-17
IL-22: Interleukin-22
IL-23: Interleukin-23
IL-12p70: Interleukin-12p70
iNOS: Inducible nitric oxide synthase
KC: Keratinocyte
LC: Langerhans cell
MCP-1: Monocyte chemoattractant protein-1
MS: Multiple sclerosis
NK cells: Natural killer cells
NF-κB: Nuclear transcription factor NF-kappa β
NO: Nitric oxide
OX: Oxazolone
PGE2: Prostaglandin E2
RA: Rheumatoid arthritis
SCFA: Short chain fatty acid
SI: Soy isoflavones
SLE: Systemic lupus erythematosus
SS: Soy saponins
T1D: Type 1 diabetes
TGF-β: Transforming growth factor β
TNF-α: Tumor necrosis factor α
TNP-Cl: Trinitrophenyl chloride
Treg: Regulatory T cells
VDR: Vitamin D receptor
XOS: Xyloligosaccharide
